# Coreference resolution improves extraction of Biological Expression Language statements from texts

**DOI:** 10.1093/database/baw076

**Published:** 2016-07-02

**Authors:** Miji Choi, Haibin Liu, William Baumgartner, Justin Zobel, Karin Verspoor

**Affiliations:** ^1^Department of Computing and Information Systems, the University of Melbourne; ^2^National ICT Australia (NICTA) Victoria Research Laboratory, Parkville, Victoria, Australia; ^3^NCBI, Bethesda, MD, USA; ^4^University of Colorado School of Medicine, Aurora, CO, USA

## Abstract

We describe a system that automatically extracts biological events from biomedical journal articles, and translates those events into Biological Expression Language (BEL) statements. The system incorporates existing text mining components for coreference resolution, biological event extraction and a previously formally untested strategy for BEL statement generation. Although addressing the BEL track (Track 4) at BioCreative V (2015), we also investigate how incorporating coreference resolution might impact event extraction in the biomedical domain. In this paper, we report that our system achieved the best performance of 20.2 and 35.2 in F-score for the full BEL statement level on both stage 1, and stage 2 using provided gold standard entities, respectively. We also report that our results evaluated on the training dataset show benefit from integrating coreference resolution with event extraction.

## Introduction

Biological networks such as gene regulatory networks, signal transduction pathways and metabolic pathways capture a series of protein-protein interactions, or relationships between proteins and chemicals, which could explain complex biological processes underlying specific health conditions. Since the scientific literature contains knowledge about relationships and events involving biomolecular entities such as proteins, genes, and chemicals, many text mining approaches have been developed for automatic information extraction from the literature (1–3). There is also much interest in standard representations of biological networks, such as the Biological pathway exchange language (4), the Systems Biology Markup Language (5) and the Biological Expression Language (BEL) (6). Such representations in a structured syntax can support not only visualisation of biological systems, but also computational modelling of these systems (7–9).

**Figure 1.**
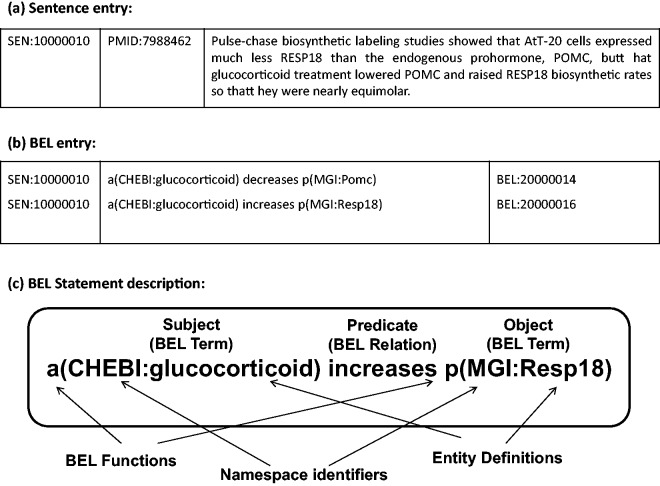
(a) Sample sentence from the BEL Track training corpus. **(b)** BEL statements corresponding to the sample sentence. **(c)** Representation of BEL statement derived from the sample sentence (a). The BEL statement describes that the abundance of chemical compound designated by ‘glucocorticoid’ in the CHEBI namespace increases the abundance of protein designated by ‘Resp18’ in the MGI namespace.

The BioCreative V Track 4 (BEL track) addressed the task of extraction of causal network information in terms of the BEL representation, a formalised representation language for biological expression (10). The BEL statements represent knowledge of relationships between biomolecular entities. BEL statements can express biological relationships, such as protein–protein interaction, or other relations between biological processes and disease stages. The BEL structure is described in detail in ‘BEL statements and dataset’ section. Two subtasks were organised in the BEL track: generation of the corresponding BEL statement for the given text evidence (Task 1), and identification of at most 10 textual evidences for a given BEL statement (Task 2). For Task 1, systematically selected sentences from publications are provided (11), and it is required to automatically generate the BEL statements corresponding to each sentence (see Figure 1). The BEL track aims to stimulate development of tools that recognise biological events, and produce BEL statements for those events. The work described in this article addresses BEL Task 1.

There has been significant progress in event extraction from the biomedical literature in recent years through targeted tasks such as BioNLP-ST (12–14) and BioCreative PPI tasks (15,16). However, extraction of complex and hidden events involving genes and proteins remains a particular challenge due to the use of coreference expressions in texts (17). Coreference expressions such as pronouns (e.g. ‘it, they’), and definite noun phrases (e.g. ‘the protein, these genes’) are one of the major obstacles for existing methods, limiting the scope of most biomedical information extraction systems to individual sentences that directly mention entities (18–20). Abundant anaphoric mentions are used to refer to biomolecular entities that were previously mentioned in the same text, such as when interactions or events are described across clauses of sentences. With the identification of these hidden relationships, coreference resolution can benefit literature-based event extraction. Hence, we hypothesised that resolving references could improve performance on the BEL statement extraction task.

To address Task 1, therefore, we developed a pipeline system which consists of the Turku Event Extraction System (TEES) (21), coupled with a coreference resolution component and an automatic system for generating BEL statements that has not previously been formally evaluated (22). We incorporate a simple rule-based coreference resolution system developed for the biomedical domain (23). In this article, we describe our pipeline in detail, introduce a strategy for mapping from BioNLP-ST event types to BEL functions, and report the overall performance of our approach in the BEL track (24). Among five participating teams, our submissions achieved the highest F-score at the full statement level for Task 1 (25). We also present our investigation of how incorporating coreference resolution impacts the performance of event extraction for the BEL track.

## Background

There have been community-wide efforts targeting biomedical event extraction since 2009, in a series of evaluations known as the BioNLP Shared Tasks (12, 26, 27). The initial task in 2009 mainly focused on extraction of biomedical events involving genes and proteins. Events were represented in terms of their type, trigger expressions, arguments and roles of arguments; analysis was based on event annotations in the GENIA journal abstract corpus (12). The scope of the task was extended to full journal documents from journal abstracts in 2011 (13). A coreference resolution subtask was incorporated in 2013, but the coreference task was not attempted by any participating teams (14). For the GENIA event extraction shared task, a state-of-the-art system (TEES) using machine learning methods achieved the best performance in the task 2009, and also achieved robust performance in 2011 and 2013 (14, 21, 28).

Text mining approaches enable the automatic extraction of such relationships from biological text. A pipeline system combining text-mining modules such as TEES and a gene normalisation component was previously implemented for event extraction and normalisation over large-scale resources (29). That system is limited to identifying events within a single sentence, and does not consider coreference resolution.

The BEL was originally developed by Selventa, a personalised healthcare organisation, with the goals of providing a formalised representation of biological relationships captured from scientific journal articles, and of supporting computational applications. To date, BEL has been used primarily in manual curation tasks; however, such manual effort cannot scale to the vastness of the biomedical literature (30). Indeed, Liu *et al.* (22) previously sought to address this by introducing a system for automatic generation of BEL statements from the biomedical literature. It uses the TEES system (21) for extraction of biological events, and translates the extracted events into BEL statements. However, the performance of the system was not formally evaluated in that prior work. Our pipeline for the BEL track is built on this system and we present its first public evaluation.

There have been several efforts addressing coreference resolution for the biomedical literature, though it remains an underexplored problem. The Protein Coreference shared task (20, 31) was organised to identify anaphoric coreference links involving proteins and genes, as a supporting task in the BioNLP shared task 2011 (27). The best performing system (32) modified an existing system, Reconcile (33), and achieved 34.1 F-score, with 73.3 Precision and 22.2 Recall. There are recent studies for biomedical coreference resolution, afterwards the BioNLP task 2011. Miwa *et al.* (34) developed a novel coreference resolution system using a rule-based approach, and improved the performance on the same gold standard corpus, reporting a 55.9 F-score. A coreference resolution module was incorporated into an existing event extraction system, EventMine (19). In that work, the output of the coreference resolution system was used as additional features for event extraction. The incorporation of the coreference resolution slightly improved event extraction performance. A hybrid approach combining rule-based and machine learning-based methods has been employed for biomedical coreference resolution (35, 36). D’Souza and Ng (36) used the combined approach for both mention detection and anaphora resolution. Li *et al.* (35) also used the combined approach for some types of anaphoric mentions; they use both rule-based and machine learning methods for relative pronoun resolution, while exclusively rule-based approaches are applied for resolution of non-relative pronouns and definite noun phrases. Those recent works show that the use of different approaches in terms of anaphora types achieved substantial improvement comparing to the best performing system in the BioNLP task 2011. However these coreference resolution systems are not publicly available. In prior work (37), a general domain coreference system (38) was evaluated on biomedical text and compared to a biomedical domain-specific system (21); the results show that domain knowledge can help coreference resolution in the biomedical domain, reporting an F-score of 37% for the biomedical domain-specific system, and an F-score of 2% for the general system.

## Methods

### BEL statements and dataset

For the BEL track at BioCreative V, sample and training datasets were provided to support system development (11). The training dataset contains 6358 sentences selected from 3052 PubMed journal articles, and 11 072 BEL statements annotated from these sentences. A sample sentence and its corresponding BEL statements are shown in Figure 1a and b. Each BEL statement is represented as a triple structure of ‘subject-predicate-object’, where subjects and objects are biomolecular entities such as proteins, genes and chemicals with namespace identifiers and their functions, and predicates describe the relationship between these entities. An example BEL statement is shown in Figure 1c. A test dataset was released for evaluation of system performance. It contains 105 sentences from 104 PubMed journal articles in the same format as the training dataset.

BEL statements capture relationships between entities (BEL Terms), making use of external vocabularies and ontologies to represent entities, including namespaces to unambiguously represent entities. Over 20 different namespaces are defined for BEL statements, for simplicity the BEL track is limited to only six namespaces to express entity types such as genes, diseases, chemicals and biological processes. The namespaces with their associated functions and occurrence counts in both training and test datasets are described in Table 1. For 11 072 BEL statements in the training data, BEL terms are mostly annotated with human protein coding genes and mouse genes.

**Table 1.** BEL abundance functions (http://wiki.ope nbel.org/display/BIOC/BEL+Documentation#BELDocumentation-Function sassociated toNamespaces) selected in the BEL track at BioCreative V

Name space	Entity concept	Function Long Form	Function Short Form	Example	Count (Train)	Count (Test)
HGNC	Human protein coding genes	proteinAbundance(),	p(),	p(HGNC:MAPK14)	7, (33%)	127 (43%)
		geneAbundance(),	g(),			
		rnaAbundance(),	r(),			
		microRNAAbundance()	m()			
MGI	Mouse genes	proteinAbundance(),	p(),	p(MGI:Mapk14)	12 231 (53%)	111 (38%)
		geneAbundance(),	g(),			
		rnaAbundance(),	r(),			
		microRNAAbundance()	m()			
EGID	Genes in a wide range of species	proteinAbundance(),	p(),	p(EGID:1432)	140 (0.6%)	0
		geneAbundance(),	g(),			
		rnaAbundance()	r()			
GOBP	Biological processes	biologicalProcess()	bp()	bp(GOBP:"cell proliferation")	1927 (8%)	23 (8%)
MESHD	Diseases	pathology()	path()	path(MESHD:Hyperoxia)	244 (1%)	11 (4%)
CHEBI	Chemicals	abundance()	a()	a(CHEBI: lipopoly-saccharide)	875 (3.8%)	23 (8%)

In addition to the abundance functions, five selected functions that describe activities such as modification, transformation or translocation are also in scope for the BEL statements in the BioCreative BEL tasks. BEL terms are arguments of these functions as described in Table 2. In the training dataset, there are 1351 entities that have a modification activity, and 205 entities for degradation activities.

**Table 2.** Other BEL functions (http://wiki.openbel.org/display/BIOC/BEL+Documentation#BELDocumentation-OtherFunctions) selected in the BEL track at BioCreative V

Function	Type	Example	Count (Train)
complex()	complex abundance	(complex(p(MGI:Itga8),p(MGI:Itgb1))) -> bp(GOBP:"cell adhesion")	758
pmod()	protein modification	p(MGI:Cav1,pmod(P)) -> a(CHEBI:"nitric oxide")	1,351
deg()	degradation	p(MGI:Lyve1) -> deg(a(CHEBI:"hyaluronic acid"))	205
tloc()	translocation	a(CHEBI:"brefeldin A") -> tloc(p(MGI:Stk16))	101
act()	molecular activity	complex(p(MGI:Cckbr),p(MGI:Gast)) -> act(p(MGI:Prkd1))	124

### System description

**Figure 2.**
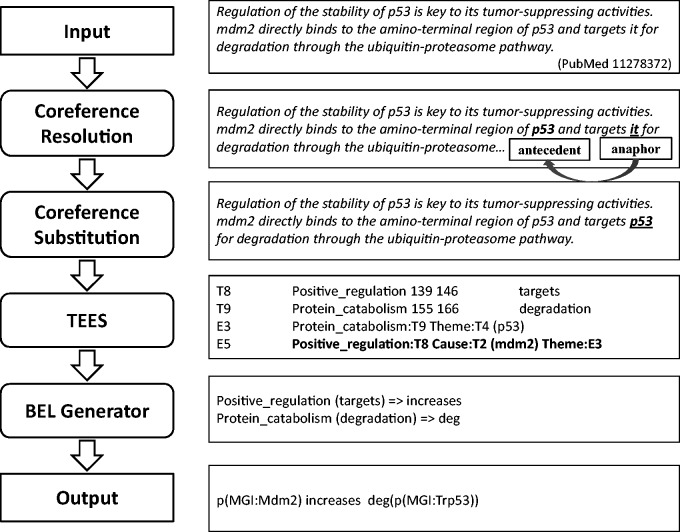
Workflow of our system for producing BEL statements from input text with examples.

Our system consists of four components in a pipeline: coreference resolution, coreference substitution, biomedical event extraction and BEL statement generation, as illustrated in Figure 2.

Input sentences are processed to identify coreference relations between anaphoric expressions and their referring mentions (antecedents). Those coreference expressions are replaced with their antecedents in the original sentences to produce resolved versions. Then, the coreference-substituted sentences are submitted to an event extraction system, TEES (21), and results of the TEES system are post-processed and converted into BEL statements. Gene and protein entities identified by the event extraction system are also normalised using selected resources such as HUGO Gene Nomenclature Committee (HGNC) Entrez Gene Identifier (EGID), and MGI in the process of generating BEL statements. In this way, we aim to identify events involving biological entities, including those that are described linguistically using anaphoric coreference mentions. The details of each component of the system are described in the following sections.

### Coreference resolution

The coreference resolution system was developed using a rule-based approach, tailored to the requirements of the BioNLP-ST’11 Coreference corpus (20). The coreference resolution system selects anaphoric mentions in the text (anaphor), and determines what the anaphor refers to (antecedent). The system consists of three stages: data pre-processing, identification of anaphoric mentions and determination of antecedents. In the pre-processing step, input texts are tokenised and syntactically parsed using the Stanford parser (39), and biomedical entities such as genes and proteins are identified using a biomedical Named Entity Recognition (NER) module, BANNER (40). Then, anaphoric mentions such as pronouns, e.g. ‘it’, ‘its’ and ‘they’, and definite noun phrases containing domain-specific nouns, such as ‘the protein’ and ‘these genes’ are identified in the step of anaphor selection. All noun phrases are considered as antecedent candidates. These candidates are ranked by a set of syntactic and semantic rules, and the top ranked candidate is determined as the antecedent corresponding to an anaphoric mention in the step of antecedent determination. The three basic rules used for the determination of an antecedent are stated below.

**Rule 1**: Antecedent candidates which do not agree in number (single or plural) with an anaphor are filtered out.

**Rule 2**: If the anaphor is a definite noun phrase, only antecedent candidates identified as genes and proteins using a biomedical NER module are kept; all others are removed.

**Rule 3**: The closest candidate that satisfies the two previous constraints is chosen.

The syntactic rule (Rule 1) used in our coreference resolution system was adapted from the approach of the Stanford general English coreference system, which links pronominal coreference mentions to their corresponding antecedents (41), while the semantic rule (Rule 2) has been motivated by the approach of Nguyen *et al.* (42). Protein and gene entities identified by BANNER, and noun phrases containing such entities are preferentially considered as antecedents for the definite noun phrases containing domain-specific terms such as ‘gene’, ‘protein’, ‘receptor’ and ‘molecule’. Even though performance of the simple coreference resolution system could not reach state-of-the-art systems such as Miwa *et al.* (34), D'Souza and Ng (36) and Li *et al.* (35), it outperforms the best published results for the BioNLP’11 Protein Coreference shared task, as shown in Table 3. We use our simple coreference system, since those systems are not publicly available. More details and an evaluation of this system are available in Choi *et al.* (23), and the system will be investigated for further improvement as future work.

**Table 3.** Our coreference resolution system performance comparing with the best performing system (33) in the BioNLP-ST’11 Coreference task (20) and state-of-the-art coreference resolution systems (italicised)

	Precision	Recall	F-score
UUtah (33)	73.3	22.2	34.1
Our system (44)	46.3	50.0	48.0
*Miwa et al.* (35)	62.7	50.4	55.9
*D’Souza and Ng* (37)	67.2	55.6	60.9
*Li et al.* (36)	67.5	69.8	68.1

Results are based on the Test data of the BioNLP’11—Protein Coreference task.

### Event extraction

We employ a state-of-the-art event extraction system, TEES (21), which was the best performing system in the BioNLP-ST’09 GE task (12). The system uses a Support Vector Machine to train a model with the GENIA corpus. In general, the TEES system takes biomedical texts as input, and has several preprocessing steps, such as sentence segmentation using GENIA Sentence Splitter (43), biomedical NER using BANNER (40), parsing texts using the BLLIP parser (44) and the Stanford parser (39), and finding head words. Then, the system identifies events involving identified entities based on a machine learning model for event detection. For our BEL track system, texts altered by the coreference substitution step are submitted to the TEES system as input. Biological events were identified using the TEES GE11 model, trained with the BioNLP-ST’11 GE corpus (27).

### Generation of BEL statements

To generate BEL statements, we adopt a system developed by Liu *et al.* (22), which converts events extracted by the TEES system into BEL statements. This BEL generation system makes use of probabilities of triggers and event arguments provided by the TEES system to compute a confidence score for each extracted event, and then translates the events from BioNLP event types into BEL statements.

Table 4 describes BioNLP event types and their corresponding BEL functions with mapping examples. For example, the BioNLP event, ‘Protein_catabolism:degradation Theme: p53’ is extracted by the TEES system from the sentence ‘mdm2 directly binds to the amino-terminal region of p53 and targets it for degradation through the ubiquitin-proteasome pathway’ as described in Figure 2, and this event is converted into ‘deg(p53)’, using the BEL function for degradation. Other BioNLP event types such as ‘positive_regulation’ and ‘negative_regulation’ are converted to BEL statements by relating and nesting occurrences of simpler event types. For example, the TEES output, ‘Positive_regulation (targets) Cause:mdm2’ in Figure 2 is converted into the BEL statement ‘p(MGI:Mdm2) increases’, since the term ‘targets’ is included in the predefined positive triggers. On the other hand, the TEES result, ‘Negative_regulation (down-regulator) Cause:IL-4 Theme:C3a’ is converted to the BEL statement ‘p(HGNC:IL4) decreases p(MGI:C3ar1)’. This is because the term ‘down-regulator’ is the one of negative trigger mentions predefined in the system. In addition, the trigger expression ‘activation’ for the event type ‘Positive_regulation’ is converted to the BEL function ‘act’, used to describe molecular activities in BEL, and its example is shown in Table 4. The event type ‘Regulation’ is not considered in the system due to its inherent ambiguity.

**Table 4.** Mapping the BioNLP event types into BEL functions

BioNLP	BEL function	BEL function type	Mapping example
Binding	p()	complex abundance	‘…*binding of several BMPs*…’ = > p(BMP-6)
Gene expression	r()	rna abundance	‘…*B cells induces both Id2 and Id3 expression…*’ = > *r*(Id1)
Localization	tloc()	translocation	‘…co-Smad (Smad4) and are translocated into the nucleus…’ = > *tloc*(Smad4)
Phosphorylation	pmod(P)	phosphorylation	‘…the *phosphorylation* level of the PPARalpha…’ = > (PPARalpha, *pmod(P)*)
protein catabolism	deg()	degradation	‘…p53 and targets it for *degradation…’* = > *deg*(p53)
Transcription	r()	rna abundance	‘…High BMP-6 *mRNA expressio*n in DLBCL…’ = > *r*(BMP-6)
*activation in* *positive_regulation*	*act()*	molecular activity	‘…IFN7 in the *activated* MMP12-treated samples…’ = > *act*(MMP12)

### BioEntity normalisation

In the process of generating BEL statements, a protein normalisation component embedded in the Liu *et al.* (22) system normalises protein mentions into concepts in the Protein Ontology (45). Preliminary work suggested the coverage provided by the protein ontology was insufficient. For protein mentions not covered in the Protein Ontology, our system searches the mentions through the fields of symbol, synonyms, alternative names and description in the resources of HGNC and MGI using an exact string matching approach. Protein mentions that could not be normalised using the Protein Ontology, HGNC and MGI resources were excluded. Error analysis suggests that these excluded mentions may be related to other concepts such as disease (MeSH Diseases) and chemical compounds (ChEBI).

## Results

### Evaluation

**Figure 3.**
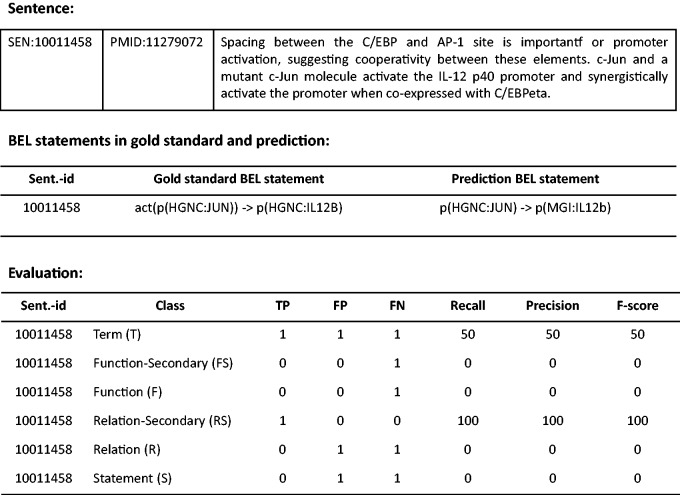
Example of an evaluation taken from the web interface. BEL statements in gold standard and system prediction are shown for the example sentence. The evaluation scores are provided for all levels.

The standard evaluation metrics consisting of Precision (the percentage of responses the system returns that are correct), Recall (the percentage of correct responses that are returned) and F-score (the harmonic mean of Precision and Recall) are used to evaluate system results at the levels of BEL terms, BEL functions, BEL relationships and the full BEL statements, separately. The function and relationship levels are also partially evaluated in what is referred to as the Secondary mode. Since the evaluation web interface is provided at (http://bio-eval.scai.fraunhofer.de/cgi-bin/General_server.rc), participants can check correctness of their system predictions. Once BEL statements that a system predicts are submitted, the result is evaluated on each level. An example of an evaluation is described in Figure 3.

At the Term level (T), a true positive (TP) is an entity the system identified correctly. It must match precisely, including abundance functions (see Table 1) as well as associated namespaces and the corresponding resource identifier to a gold standard entity. Identified entities that do not match with a gold annotation are defined as false positives (FP). Entities annotated in the gold standard datasets which are missed by the system are defined as false negatives (FN). As shown in Figure 3, for instance, the term ‘p(HGNC:IL12B)’ in gold standard is a FN, since the system predicted ‘p(MGI:IL12b)’ instead, while that prediction is a FP. At the Function level (F), abundance functions and activity functions e.g. ‘deg’, or ‘act’, are evaluated (see Table 2). If an activity function plus the correct abundance function in the argument matches, it is considered a TP. At the Secondary Function level (Fs), the main function alone is assessed, ignoring the namespace of the entity. For example, the activity function, ‘act’, is missed by the system in the example evaluation. As a result, the result is a FN at both Function and Secondary Function levels. At the Relationship level (R), the relationship between entities (subject and object) is evaluated. TPs are defined as relationships the system returned where a relationship between a subject and an object is correct. On the other hand, partial matches for relationships are evaluated at the Secondary Relationship level (Rs). Cases of partial relationships include a correct relationship with an incorrect subject and a correct object, a correct relationship with a correct subject and an incorrect object, and an incorrect relationship with a correct subject and a correct object; these are scored as TPs at the Secondary Relationship level. For the overall evaluation, each BEL statement (S) is evaluated if it is correct and complete at the full BEL statement level.

### Results for Task 1

We report the official results of our submitted runs on the test dataset in Table 5. Results are reported for Runs 1–3 in Stage 1 of BEL track Task 1. Each run used a different approach, as follows:

**Table 5.** Official results on test data for BEL task 1 in Stage 1

		TP	FP	FN	P	R	F
Run 1 (without coref.)	Term	64	12	236	84.2	21.3	34.0
	Function Second.	3	1	53	75.0	5.4	10.0
	Function	3	1	63	75.0	4.6	8.6
	Relation-Second.	54	5	148	91.5	26.8	41.4
	Relation	32	21	170	60.4	15.8	25.1
	Statement	25	21	177	54.4	12.4	20.2
Run 2 (with coreference)	Term	64	15	236	81.0	21.3	33.8
	Function Second.	4	1	52	80.0	7.1	13.1
	Function	3	2	63	60.0	4.6	8.5
	Relation-Second.	54	8	148	87.1	26.7	40.9
	Relation	32	24	170	57.1	15.8	24.8
	Statement	25	24	177	51.0	12.4	19.9
Run 3 (with coreference and extended BEL function)	Term	64	15	236	81.0	21.3	33.8
	Function Second.	5	1	51	83.3	8.9	16.1
	Function	3	4	63	42.9	4.6	8.2
	Relation-Second.	54	8	148	87.1	26.7	40.9
	Relation	32	26	170	55.2	15.8	24.6
	Statement	25	26	177	49.0	12.4	19.8

Run 1, an approach without coreference resolution; Run 2, an approach with coreference resolution; Run 3, a coreference approach with extended BEL function.

**Run 1** consists of the basic TEES + BEL mapping system, with no coreference resolution step;

**Run 2** uses the complete pipeline, including coreference resolution;

**Run 3** extends the system in complete pipeline of Run 2 with an additional BEL function, *act*(), as described in Table 4.

Our system achieved an F-Score of 20.2, with Precision 54.4 and Recall 12.4 at the full Statement level in Run 1. Incorporating coreference resolution (Run 2) increased system performance of F-score from 10.0 to 13.1 at the secondary function level comparing to Run 1, but slightly decreased performance at other levels. This is because the number of coreference mentions is small in the test dataset as described further in ‘Comparison of performance with coreference resolution’ section, and the coreference approach produced more FPs than without coreference. Due to the small number of coreference mentions in the test dataset, we use the training dataset as a more rigorous evaluation of system performance with and without the coreference resolution component. These evaluation results are presented in ‘Comparison of performance with coreference resolution’ section. Note that since our method does not use this data in any way for supervision, this is a valid evaluation strategy.

In a second test phase (Stage 2), gold standard entities for the test dataset were given by the BioCreative BEL task organisers in order to allow the analysis to focus on the task of event extraction, rather than the task of named entity recognition. This is therefore an “oracle” scenario, where the event extraction step is seeded with perfect information about entities. We provided the gold standard entities as input to the extended system corresponding to Run 3 in Stage 1, and the results are described in Table 6. When compared with Run 3 in Stage 1, the use of gold standard entities resulted in substantially improved system performance, with an absolute increase of F-score (33.8 vs. 54.3), (8.2 vs. 20.8), (24.6 vs. 43.7) and (19.8 vs. 35.2) at the Term, Function, Relation and Statement levels, respectively.

**Table 6.** Results on test data for BEL task 1 in the Stage 2

		TP	FP	FN	P	R	F
*NonCoreference	Term	101	5	199	95.3	33.7	49.8
	Function Second.	8	2	48	80.0	14.3	24.2
	Function	7	1	59	87.5	10.6	18.9
	Relation-Second.	84	3	118	96.6	41.6	58.1
	Relation	57	16	145	78.1	28.2	41.5
	Statement	44	18	158	71.0	21.8	33.3
Coreference	Term	113	3	187	97.4	37.7	54.3
	Function Second.	9	4	47	69.2	16.1	26.1
	Function	8	3	58	72.7	12.1	20.8
	Relation-Second.	91	3	111	96.8	45.1	61.5
	Relation	62	20	140	75.6	30.7	43.7
	Statement	48	23	154	67.6	23.8	35.2

Coreference, a coreference approach with extended BEL function using the given gold standard entities, NonCoreference, an approach without coreference resolution with extended BEL function using the given gold standard entities.

To directly assess the impact of coreference resolution in this scenario, we ran a variant of the system without the coreference module but in the oracle condition (See the NonCoref section of Table 6; note that this system was not included in the official results; these results were generated for the test data after the end of the shared task). In contrast to Stage 1, the performance when incorporating coreference resolution is slightly higher than without coreference in Stage 2. The coreference approach produced more outputs overall. This included not only TPs, but also more FNs than the approach without coreference resolution. Overall, there was a slight performance improvement attributable to coreference resolution over the test data in the oracle condition. (NB: The result of *NonCoref. was not submitted to the BEL task, but the evaluation was conducted later as a subsequent experiment using the official test data).

### Comparison of performance with coreference resolution

Based on a co-reference analysis framework that classifies coreference mentions by their types, and considers the broader syntactic and semantic characteristics of coreference links (46), we analysed the gold standard datasets by categorising types of coreference expressions. The analysis of mention types appears in Table 7. There are 257 personal pronouns (e.g. ‘it, they’), 411 possessive pronouns (e.g. ‘its, their’) and 507 definite noun phrases (e.g. ‘the protein, these genes’) in the training dataset, while only six personal pronouns and five possessive pronouns in the test dataset. Relative pronouns such as ‘which’ and ‘that’ were not addressed in this task, since the coreference system had a negative impact on event identification for these pronouns. This was determined based on an investigation on the training dataset that demonstrated quantitatively that the resolving relative pronouns degraded performance (results not reported in this article).

**Table 7.** Statistics of anaphor types in the gold standard dataset at the BioCreative V shared task Track 4 (BEL track)

Anaphor type	Training dataset		Test dataset	
	Numbers	Sentence prop.	Numbers	Sentence prop.
Relative pronoun	1313	21%	14	13%
Personal pronoun	257	4%	6	6%
Possessive pronoun	411	6%	5	5%
Definite noun phrase	507	8%	0	–
Total	2488		25	

Numbers are counts of occurrence of each anaphoric type, and Sentence prop. is the percentage of all sentences that include at least one anaphor of relevant type.

We compare our system performance with and without the coreference resolution component on the training dataset in terms of the types of coreference links defined by the analysis framework (46) in Table 8 which allows for a fine-grained analysis of information extraction impacted by different types of coreference. Since no component in the pipeline makes use of the provided training data for development, but rather was developed independently of the BEL task as described in ‘Methods’ section, we are able to use all training data as test data. Only 709 sentences that contain anaphoric expressions in the training data were used for this evaluation. Performance is reported in terms of anaphor types, and at the levels of Term, Function, Fs, Relation, Rs and Statement using the evaluation interface^1^ provided for the BEL track.

**Table 8.** Comparison of performance between an approach with coreference resolution and an approach without it on anaphoric sentences in the training dataset, in terms of anaphor types

		Without Coreference						With Coreference					
		TP	FP	FN	P	R	F	TP	FP	FN	P	R	F
Pers. pronoun	T	34	45	89	43.0	27.6	33.7	55	43	68	56.1	44.7	**49.8**
	Fs	2	4	44	33.3	4.4	7.7	6	8	40	42.9	13.0	**20.0**
	F	2	4	58	33.3	3.3	6.1	6	10	54	37.5	10.0	**15.8**
	Rs	25	22	54	53.2	31.7	39.7	44	24	35	64.7	55.7	**59.9**
	R	5	46	74	9.8	6.3	7.7	16	46	63	25.8	20.3	**22.7**
	S	2	48	77	4.0	2.5	3.1	5	55	74	8.3	6.3	**7.2**
Poss. pronoun	T	82	74	125	52.6	39.6	45.2	100	74	107	57.5	48.3	**52.5**
	Fs	20	12	75	62.5	21.1	31.5	23	9	72	71.9	24.2	**36.2**
	F	13	24	116	35.1	10.1	15.7	17	18	112	48.6	13.2	**20.7**
	Rs	76	33	74	69.7	50.7	58.7	89	31	61	74.2	59.3	**65.9**
	R	27	81	123	25.0	18.0	20.9	34	79	116	30.1	22.7	**25.9**
	S	13	85	137	13.3	8.7	**10.5**	12	87	138	12.1	8.0	9.6
Def. NP	T	27	22	45	55.1	37.5	44.6	36	26	36	58.1	50.0	**53.7**
	Fs	9	3	22	75.0	29.0	41.9	11	3	20	78.6	35.5	**48.9**
	F	4	10	38	28.6	9.5	14.3	10	5	32	66.7	23.8	**35.1**
	Rs	26	5	23	83.9	53.1	65.0	30	10	19	75.0	61.2	**67.4**
	R	10	20	39	33.3	20.4	25.3	16	26	33	38.1	32.7	**35.2**
	S	3	23	46	11.5	6.1	8.0	7	29	42	19.4	14.3	**16.5**
ALL	T	141	140	255	50.2	35.6	41.7	188	143	208	56.8	47.5	**51.7**
	Fs	30	18	139	62.5	17.8	27.7	39	19	130	67.2	23.1	**34.4**
	F	18	37	209	32.7	7.9	12.8	32	32	195	50.0	14.1	**22.0**
	Rs	126	60	147	67.7	46.2	54.9	162	65	111	71.4	59.3	**64.8**
	R	42	146	231	22.3	15.4	18.2	65	151	208	30.1	23.8	**26.6**
	S	18	155	255	10.4	6.6	8.1	23	171	250	11.9	8.4	**9.9**

The higher F-score (with vs. without coreference) is indicated in bold.

Overall, system performance improves when incorporating coreference resolution. When considering the resolution of personal pronouns, our system improved Precision, Recall and F-score at each level. We observe an absolute increase in Precision from 43.0 to 56.1, in Recall from 27.6 to 44.7 and in F-score from 33.7 to 49.8 at the Term level. The inclusion of coreference resolution for definite noun phrases also resulted in improvement of Precision (28.6 vs. 66.7), Recall (9.5 vs. 23.8) and F-score (14.3 vs. 35.1) at the Function level (Pers., Personal; Poss., Possessive; NP,  Noun Phrase; ALL, Sum of Per. Pronoun; Poss. Pronoun and Def. NP; T, Term level; Fs, Function-Secondary level; F, Function level; Rs, Relation-Secondary level; R, Relation level; S, full Statement level)

## Discussion

The task of extraction of biomolecular relationships in the form of BEL statements is highly complex. The task requires identification of entity types, and disambiguation of entities including namespaces and their roles, as well as correct identification of activity status and relationships between entities. Even though there were simplifications made for the shared task, such as restricting namespaces to 6 of the 20 namespaces used in the full BEL specification, an acceptance of orthologous identifiers for HGNC, MGI and EGID namespaces, and a tolerance of simplified statements (e.g. ‘act()’ allowed for ‘kin(), tscript()’ and ‘cat()’), the five participating systems achieved low performance for the full statement level as described in Table 9. Our system (S3) achieved the best F-score of 20.2%, and system S4 and S5 achieved slightly lower F-score. System S4 achieved much lower F-score of 2.7% at the Function level, which reduced the system precision at the full statement level, even though achieved higher F-score at the Term and Relation levels than our system. System S5 also achieved lower Precision at the full statement level, even though performed the best F-score at the levels of Term, Function and Relation, with our system limited largely by Recall. The system S4 (47) used different approaches for each subtask, e.g. a hybrid (Conditional Random Fields and dictionary lookup) approach for identification of entities and abundance functions, a rule-based approach for entity normalisation, and a statistical parser for classification of relationships. The system S5 (48) used existing systems such as PubTator (49) and BeCAS (50) for identification of biomedical concepts, a dictionary lookup method for entity normalisation and a rule-based approach for extraction of biological events.

**Table 9.** Evaluation results of participating systems for Task 1

	Term			Function			Relation			Full statement		
System	P	R	F	P	R	F	P	R	F	P	R	F
S1	38.0	28.3	32.4	26.3	7.6	11.8	1.2	1.5	1.3	0.8	1.0	0.9
S2	52.6	60.3	56.2	11.2	18.2	13.9	9.7	8.4	9.0	7.6	6.4	7.0
S3 (ours)	84.2	21.3	34.0	75.0	4.6	8.6	60.4	15.8	25.1	54.4	12.4	20.2
S4 (46)	64.2	61.0	62.6	12.5	1.5	2.7	39.6	19.8	26.4	31.2	14.4	19.7
S5 (47)	82.0	59.3	68.9	30.7	34.9	32.6	69.4	38.1	49.2	26.4	13.9	18.2

The best F-score among their submissions is described for each system; adapted from Fluck *et al.* (25).

When incorporating coreference resolution, system performance on the training and the test datasets differed substantially. The evaluation results on the training dataset show that the coreference resolution approach markedly improved system performance compared with the result without coreference resolution as shown in Table 8. On the other hand, the approach with coreference resolution slightly reduced system performance on the official test dataset in Stage 1, producing additional FPs (Run 1 and Run 2 in Table 5). However, the test data are small and contains few instances of coreference. There are only 11 coreference relations (personal and possessive pronouns only considered) in 105 sentences in the test dataset, as summarised in Table 7. This small number of coreference mentions in the test data is insufficient to evaluate the impact of coreference resolution. Our system produced four additional BEL statements over the test data with coreference resolution, as compared to the result without coreference resolution. These statements are all FPs due to system errors in normalisation of entity mentions to IDs, and in identification of events involving entity types other than proteins and genes. We discuss the impact of coreference resolution on event extraction further in ‘impact of coreference resolution’ section.

### Error analysis

The BEL task requires identification of a range of entity types including genes, diseases, chemicals and biological processes in the input texts, as described in Table 1. However, our system is limited to identifying events involving gene and protein entities only, due to the reliance on BANNER and its gene model for entity recognition. There are 57 diseases, chemical and biological process entities among 295 entities in the test dataset described in Table 1. Given the limitations of the system, these entities were ignored; no BEL statements involving them could be identified.

There is a notable difference in the results between Stages 1 and 2, the oracle condition. With gold standard entities provided, our system substantially improved overall performance in Stage 2 (Table 6), indicating that improved entity detection would greatly benefit our system. We will expand the range of entity types and address relations involving these entities in future work. For instance, we may be able to build on the work of Funk *et al.* (51) to address identification of Gene Ontology and ChEBI terms and DNorm for Diseases (52).

There was also a limitation in the performance of our system stemming from which trigger mentions are used to produce BEL statements in the original BEL generation system that we employed (22). Low Recall at the Function and Function-Secondary Levels in Table 5 shows that our system failed to capture event trigger mentions associated with many BEL functions. When the original BEL generation system was developed, the trigger mentions were derived from the BioNLP’ST 09 corpus (12). As a subsequent experiment, we extended a set of trigger mentions by taking advantage of the BioNLP’ST 2011 and 2013 gold standard corpora (13, 14). However, this extension did not result in an improvement in performance, and the results of this further experiment are not presented in this paper. In future work, we will consider other methods to better address this issue.

### Impact of coreference resolution

Even though the process of coreference resolution resulted in a slight performance reduction in the final result on the test dataset, the approach has the potential to improve discovery of implied and complex biological events, as indicated by our experiments over the training data. For example, the following passage expresses a relationship between the personal pronoun ‘**It’** and the gene ***TIMP-1*** in the text.

‘Interestingly, **IL-13** did cause an ∼80% decrease in pulmonary a1-AT expression (Figure 13). **It** also caused a significant increase in TIMP-1 expression that was seen after as little as 1 day and was readily apparent with longer periods of dox administration (Figure 13, and data not shown) (*P* < 0.05 for all comparisons)’. (SEN:10028008)

Our system identifies the coreference relationship between the anaphor ‘It’ and the gene ***IL-13*** (antecedent) mentioned in the previous sentence, and automatically substitutes the pronoun with its antecedent. Consequently, the event, ‘IL13 increases TIMP1 expression’ is successfully extracted. This would not be identified without coreference resolution. In the results described in Table 8, our system including coreference resolution produced more TPs overall, e.g. 188 vs. 141 at the Term level, 32 vs. 18 at the Function level, 65 vs. 42 at the Relation level and 23 vs. 18 at the full Statement level.

We also compare the approaches with and without coreference resolution on the training dataset using a statistical significance test (paired *t*-test) in Table 10. Differences between the approaches at each evaluation level are significant at the 95% confidence interval (*Note: With Coref. performs better than Without Coref., when t-score is under [1.699, ∞), while Without Coref. performs better, when t-score is under (−∞, −1.699]. Otherwise, there is no significant difference between With Coref. and Without Coref.).

**Table 10.** Results of paired t-test between an approach with coreference resolution and an approach without it on the training dataset for each level

		Term	Function_S.	Function	Relation_S.	Relation	Statement
With coreference without coreference	t	6.82	4.77	5.20	5.51	5.79	5.34

At the 95% confidence interval (df = 29), a score of ± 1.699 indicates a significance difference; all reported differences are significant.

## Conclusions

To address the BEL task in the BioCreative V, we have developed a system for biological event extraction, targeting generation of BEL statements from the biomedical literature, by incorporating several existing text mining systems. In this task, we have also explored how a coreference resolution component can help to improve event extraction. Even though our performance on the official test data did not show a strong benefit from the incorporation of coreference resolution due to a small number of coreference instances in that data, we have demonstrated that over a larger data set, coreference resolution does significantly improve overall event extraction performance. The coreference resolution approach has the potential to discover implied relationships among entities, and thus impact event and network extraction in the biomedical domain.

The BEL task makes use of six possible namespaces for various biological entity types. However, our system is limited to identifying events involving specifically proteins and genes only and did not emphasise entity normalisation as a primary task. We report a substantial improvement in system performance using the given gold standard entities in the oracle setting of BEL Task 1, Stage 2. In future work, we will further expand the scope of named entity recognition to extract events involving other relevant biological concepts and entities, in order to achieve further improvement in our overall information extraction capability.

## Footnotes

1 http://bio-eval.scai.fraunhofer.de/cgi-bin/General_server.rc.
